# Vaccination governance in protracted conflict settings: the case of northwest Syria

**DOI:** 10.1186/s12913-024-11413-1

**Published:** 2024-09-12

**Authors:** Ronja Kitlope Baatz, Abdulkarim Ekzayez, Yasser Najib, Munzer Alkhalil, Mohammad Salem, Mohammed Ayman Alshiekh, Preeti Patel

**Affiliations:** 1 Deventer Hospital, Deventer, Netherlands; 2 Research for Health System Strengthening in northern Syria (R4HSSS), The Centre for Conflict & Health Research (CCHR), King’s College London, Strand, WC2R 2LS London, UK; 3 Syria Development Centre (SyriaDev), London, UK; 4 Syria Immunisation Group (SIG), Gaziantep, Turkey; 5 Syria Public Health Network, London, UK; 6 Research for Health System Strengthening in Northern Syria (R4HSSS), UOSSM, Gaziantep, Turkey; 7 Vascular Senior Clinical Fellow, Manchester Royal Infirmary, Manchester, UK

**Keywords:** Immunisation, Vaccination, Syria, Health governance, Conflict setting, Localisation

## Abstract

**Background:**

Effective vaccination governance in conflict-affected regions poses unique challenges. This study evaluates the governance of vaccination programs in northwest Syria, focusing on effectiveness, efficiency, inclusiveness, data availability, vision, transparency, accountability, and sustainability.

**Methods:**

Using a mixed-methods approach, and adapting Siddiqi’s framework for health governance, data were collected through 14 key informant interviews (KIIs), a validating workshop, and ethnographic observations. Findings were triangulated to provide a comprehensive understanding of vaccination governance.

**Results:**

The study highlights innovative approaches used to navigate the complex health governance landscape to deliver vaccination interventions, which strengthened sub-national vaccination structures such as The Syria Immunisation Group (SIG). The analysis revealed several key themes. Effectiveness and efficiency were demonstrated through cold-chain reliability and extensive outreach activities, though formal reports lacked detailed analysis of vaccine losses and linkage between disease outbreak data and coverage statistics. Key informants and workshop participants rated the vaccination strategy positively but identified inefficiencies due to irregular funding and bureaucracy. Inclusiveness and data availability were prioritised, with outreach activities targeting vulnerable groups. However, significant gaps in demographic data and reliance on paper-based systems hindered comprehensive coverage analysis. Digitalisation efforts were noted but require further support. The SIG demonstrated a clear strategic vision supported by international organizations such as the World Health Organization, yet limited partner participation in strategic planning raised concerns about broader ownership and engagement. While the SIG was perceived as approachable, the lack of public documentation and financial disclosure limited transparency. Internal information sharing was prevalent, but public communication strategies were insufficient. Accountability and sustainability faced challenges due to a decentralized structure and reliance on diverse donors. Despite stabilizing factors such as decentralization and financial continuity, fragmented oversight and reliance on donor funding remained significant concerns.

**Discussion:**

The study highlights the complexities of vaccination governance in conflict-affected areas. Comparisons with other conflict zones underscore the importance of local organisations and international support. The SIG’s role is pivotal, but its legitimacy, transparency, and inclusivity require improvement. The potential transition to early recovery in Syria poses additional challenges to SIG’s sustainability and integration into national programs.

**Conclusion:**

The governance of vaccination in northwest Syria is multifaceted, involving multiple stakeholders and lacking a legitimate government. Enhancing transparency, local ownership, and participatory decision-making are crucial for improving governance. The role of international bodies is essential, emphasising the need for structured feedback mechanisms and transparent monitoring processes to ensure the program’s success and sustainability.

**Supplementary Information:**

The online version contains supplementary material available at 10.1186/s12913-024-11413-1.

## Key message

• A hybrid governance model that combines top-down and bottom-up approaches effectively improves immunisation programs in conflict settings and promotes local ownership.

• In conflict settings, immunisation programmes require strong and direct intervention from the WHO with central management and coordination of the vaccine activities.

• In conflict areas, when the government is a party to the conflict or has limited access to some areas, United Nations institutions must intervene to manage or support vaccine activities in partnership with local entities, regardless of notions of national sovereignty.

• In Syria, the reluctance of United Nations institutions to fill the void left by the state in areas outside its control and provide vaccines led to the emergence of many diseases, including polio in 2013 and measles in 2017.

**Supplementary Information:**

The online version contains supplementary material available at 10.1186/s12913-024-11413-1.

## Introduction

Immunisation services are essential for any health system to ensure protection against major transmissible diseases. Armed conflicts often influence the availability, quality, accessibility, and uptake of vaccination services, which can lead to the emergence of outbreaks and epidemics [1, 2]. The restoration of regular immunisation services in emergency contexts has not been extensively studied, and protracted crises “underscore the need to consider matters beyond the emergency mindset” [3]. Furthermore, health partnerships remain largely centred on national governments [4], raising the question of how areas beyond state control can best organise routine vaccination services.

The Syrian conflict, which started in March 2011, has had a devastating impact on the health system of the country; with vaccination coverage dropping from more than 90% for the Diphtheria, Tetanus & Pertussis (DTP) vaccine pre-conflict, to less than 10% in some areas [5, 6]. With the fall of some areas under opposition control, the Syrian government began to withhold vaccinations from these areas, while simultaneously attacking healthcare facilities and infrastructure [7]. The decline in vaccine coverage resulted in outbreaks of Vaccine Preventable Diseases (VPDs), including polio (2013, 2017) and measles (2017, 2018) [8, 9]. This led to vaccination becoming a priority for the humanitarian sector following the outbreak of wild poliovirus in October 2013.

**Fig. 1:**
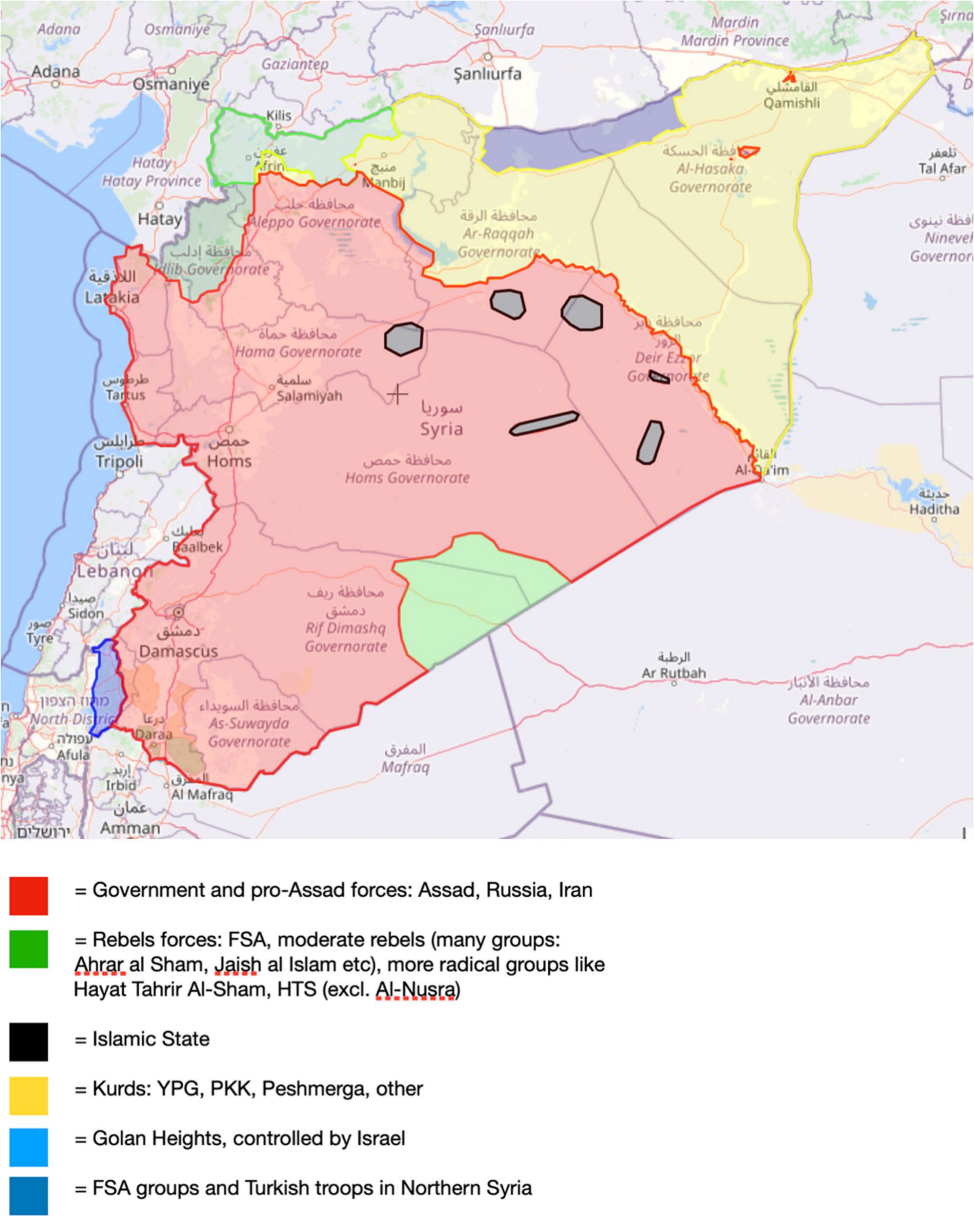
Areas of control in Syria as of April 2023. Source: Liveuamap, 2023

Syria is now roughly divided into three main areas of control: the self-administration region of northeast Syria controlled by Kurdish majority forces, the governmental areas in the central, coastal and southern regions, and various opposition forces in the northwest. These delineations are visually depicted in Fig. 1, where the regions are represented by the colours yellow, red, and green, respectively [10]. Opposition controlled areas in northwest Syria has a population of about 4.5 million people, of whom over a third, 1.8 million, live in camps, which is the area of focus in this study [11]. According to The United Nations Office for the Coordination of Humanitarian Affairs (OCHA), about 90% of the population is dependent on donor aid for their subsistence, including for health care [12]. Northwest Syria is governed by two main forces, the opposition forces with Turkish support in northern Aleppo, and Hayat Tahrir Al-Sham (HTS) in Idlib Governorate [13]. HTS is listed as a terrorist organisation by the US, UN, EU and Turkey, preventing aid organisations from working with them [14]. As there is no recognised government in northwest Syria and no clear end in sight to the conflict, international aid organisations are facing a long-term problem of coordination, particularly in programmes which require stability and effective governance, such as routine immunisation. Humanitarian access to northwest Syria has been using border crossing points from Turkey under annually renewed Resolutions by the UN Security Council since July 2014 [15]. However, this crossing became limited to only one crossing point, the Bab el Hawa border in 2019, and later this crossing has expired with the failure to renew this UN Resolution after being vetoed by Russia and China. The Security Council’s failure to reauthorise the long-standing cross border humanitarian aid mechanism in July 2023, has laid bare the implications for the humanitarian situation in Syria coupled with a deepening divide on the Security Council’s engagement on the issue. There is now uncertainty about the future of the aid mechanism and other UN operations in the region [16].

Prior to the conflict, Syria had advanced vaccination governance and high immunization coverage, with World Health Organization (WHO) and United Nations International Children’s Emergency Fund (UNICEF) estimating DTP vaccine coverage at over 89% [17]. During the conflict, vaccination activities faced significant challenges following the withdrawal of the Syrian Government from opposition-controlled territories in 2012. This led to disruptions in the supply chain, human resource shortages, and governance collapse, resulting in reduced vaccination coverage and outbreaks of diseases such as Polio and Measles [18]. Emergency vaccination campaigns were initiated by local and international actors to address these outbreaks, with the establishment of entities such as the Polio Task Force and Measles Task Force. Since 2016, vaccination efforts have been led by the Syria Immunisation Group (SIG), formed by local humanitarian actors and co-chaired by WHO and UNICEF. Please see Table 1 for the vaccination schedule in Syria before and after the conflict.

Despite Syria’s eligibility for Global Alliance for Vaccines and Immunization (GAVI) support in 2019, actual funding received remains lower than pledged, making it challenging to assess the total cost of vaccine activities [19, 20]. The literature on vaccination governance in northwest Syria is scant, with limited distinction between northwest Syria and government-controlled areas. Comprehensive accounts of SIG’s work are rare, with the WHO 2020 report on Syria providing one notable exception [21]. This lack of literature may reflect the complex political economy context, as government withholding of vaccinations prompted alternative actors to facilitate vaccination and governance [22].

**Table 1:** Vaccination schedule in Syria before and during the conflict

	Vaccination schedule prior the conflict	Vaccination schedule in NWS after the conflict
Birth	Hep B1 + BCG + OPV0	Hep B0 + BCG
3 months	Penta1 + Hep B2	Penta1 + IPV1 + OPV1
5 months	Penta2	Penta2 + IPV2 + OPV2
7 months	Penta 3 + Hep B3 + OPV 1	Penta 3 + OPV 3
1 year	MMR 1 + OPV2 + Vit A	MMR 1 + Vit A
18 months	Penta booster + MMR 2 + OPV 3 + Vit A	Penta booster + MMR 2 + OPV 4 + Vit A

This study aims to explore the effectiveness and efficiency of vaccination governance in northwest Syria (NWS), its responsiveness, inclusivity, and informed decision-making processes, as well as its vision, strategy, transparency, and accountability. By examining these aspects, the research seeks to provide a comprehensive understanding of how vaccination programs operate in conflict-affected areas and the unique challenges they face.

## Methodology

This study employed a mixed-methods approach consisting of semi-structured qualitative interviews, a validation workshop, and ethnographic observations to comprehensively investigate vaccination governance in northwest Syria.

Firstly, we adapted the Siddiqi framework for health governance [23] with modifications to accommodate the unique challenges and dynamics present in northwest Syria. Its six key principles offer a structured approach to assess governance effectiveness, inclusivity, transparency, and accountability, which were central to the study’s objectives. This adapted framework guided the data collection, analysis, and interpretation processes, providing a structured approach to examining vaccination governance from a health system perspective.

Secondly, we conducted 14 semi-structured qualitative Key Informant Interviews (KIIs) with key informants involved in vaccination governance in northwest Syria. Purposive sampling was used to select participants representing various stakeholders, including representatives from local health directorates, international organizations, and community leaders - please see Table 2. Participants were identified based on their expertise and roles in vaccination delivery. We approached potential participants through email and phone calls, explaining the purpose of the study and inviting them to participate. Those who agreed to participate were scheduled for interviews at their convenience. The semi-structured interview guide (see Supplementary Material) aimed to explore participants’ experiences, perspectives, and challenges related to vaccination governance. The interviews were audio-recorded with participants’ consent and transcribed verbatim for analysis. Thematic analysis was conducted using both deductive and inductive approaches, with the Siddiqi framework guiding the thematic grouping and coding process. Notably, only two of the interviewees identified as female. This gender disparity reflects broader gender imbalances in leadership positions within the context of conflict-affected areas and may influence the perspectives and priorities discussed during the workshop.

Thirdly, a validation workshop was conducted in Gaziantep in November 2023 to validate the findings from the interviews and gather additional insights from stakeholders. The 15 participants in the workshop included key informants who had been interviewed, as well as other relevant stakeholders – please see Table 2. An overview of the key findings per theme identified in the interviews was presented, followed by a discussion to validate and elaborate on these findings. The workshop facilitated a collaborative process to prioritize the main achievements and challenges identified in the interviews.

In addition, ethnographic observations were conducted alongside the field data collection to provide contextual insights into vaccination delivery and governance practices in northwest Syria. These observations involved daily immersion in the field, engaging in informal conversations with stakeholders, and documenting observations through field notes. This approach was used to build trust with key stakeholders, helping them understand the importance of our research and encouraging them to openly share their views and participate in research activities. The informal conversations and daily immersion provided rich qualitative data on the local context, practices, and challenges, which were crucial for interpreting the collected data. Additionally, relevant documents, such as reports and policy documents, were collected and analysed to complement the ethnographic data.

The three sets of data—interviews, workshop discussions, and ethnographic observations—were triangulated to enhance the validity and reliability of the findings. Triangulation was conducted through comparing and cross-referencing information from each data source. Initially, key themes and findings from the interviews were identified and categorised. These themes were then cross-checked against insights gathered from workshop discussions and ethnographic observations to identify common patterns, discrepancies, and unique contributions. Any discrepancies were further investigated through follow-up discussions or additional document analysis to resolve inconsistencies and confirm findings.

**Table 2:** Overview of study participants

Method	# participants	Affiliations
Semi-structured interviews	14	SIG (4), Health Cluster (1), Idlib Health Directorate (1), Partner organisations (8)
Validation workshop	15	SIG (4), Partner organisations (11)

Ethical approval was obtained from the Institutional Review Board of King’s College London (MRA-22/23-34048) and, due to the sensitive nature of the subject, anonymity of participants was deemed critical. Informed consent was signed by all interviewees and interview records were deleted within two days after the interview, with notes being de-identified. All records and code-keys were stored on a password-protected secure drive.

## Results

This section presents five key themes that emerged from the data: effectiveness and efficiency, inclusiveness and data availability, clear vision with limited participatory strategy development, limited transparency, and accountability and sustainability. For each theme, findings are triangulated from interviews, workshop discussions, and ethnographic observations to provide a comprehensive understanding of vaccination governance in northwest Syria.

### Effectiveness and efficiency

Field observations highlighted the operational success of the vaccination strategy, particularly in maintaining cold-chain reliability and conducting extensive outreach activities. Researchers noted that cold-chain facilities appeared well-maintained and outreach teams were active in various communities.

Document analysis corroborated these observations, although it revealed a lack of detailed analysis in formal reports regarding vaccine losses and linkage between disease outbreak data and coverage statistics. The annual report for 2021 noted the distribution of over 1.5 million routine vaccines and approximately 350,000 COVID-19 vaccines (SIG, 2021).

KIIs provided subjective assessments of effectiveness, with most participants rating the vaccination strategy very positively. For example, one key informant stated, “Cold-chain is very complicated, and (…) we have never faced gaps in the cold-chain. The outreach activities too, they are amazing in screening the whole community” (K-07). Another participant commented, “I think there are three successful entities in Syria. White Helmets, Early Warning and Response Network (EWARN) and SIG. Basically, they are performing governmental performance, without being a government” (K-10).

The workshop echoed these sentiments, emphasising the reliability of cold-chain logistics and the effectiveness of outreach programs. Participants highlighted the comprehensive knowledge outreach teams had about the communities, such as culture and health seeking behaviour, which facilitated high vaccine coverage.

Analysis suggests that while the subjective assessments are positive, the lack of detailed data in formal documents indicates a need for more robust quantitative evaluation mechanisms to fully substantiate these claims.

Efficiency was qualitatively explored through factors such as human resources, bureaucracy, corruption, and the non-governmental nature of the program. Field observations noted strong capacity among staff and stable governance structures.

Documents reviewed pointed to significant bureaucracy but suggested it was a necessary component to prevent corruption. KIIs reinforced this, with one participant noting, “You can’t do any humanitarian process without this paperwork, to be honest. It is the right way, because otherwise you are corrupted” (K-01). Another added that corruption was low due to the nature of the resources involved, stating, “There are few reasons for people to steal from this programme. It isn’t food baskets or money, it’s vaccines” (K-01).

Workshops confirmed these findings but also highlighted inefficiencies due to the lack of government services and irregular funding, which led to service discontinuations. One workshop participant explained, “The Expanded Programme for Immunisation (EPI) is continuous, it should be a 2 or 3 year project. For example, the first project ends by the end of May and the next project starts mid-June. So, there is a gap for staff, so they don’t receive their salaries” (W-02).

In conclusion, while the vaccination governance seems to be efficient with limited observed effectiveness, challenges remain in documentation and the impacts of funding irregularities, short termism and uncertainty.

### Inclusiveness, responsiveness, and data availability

Field observations indicated that accessibility and inclusiveness are prioritized in vaccination efforts, with outreach activities playing a crucial role in reaching vulnerable groups. Researchers observed that outreach sessions outnumbered fixed sessions, reflecting the emphasis on inclusivity.

Document analysis revealed systematic data collection efforts to identify reasons for missed vaccinations to target vulnerable groups, including zero-dose children, people with disabilities, female-headed households, and those living in remote areas. However, significant gaps in demographic data and reliance on paper-based systems were noted, hindering comprehensive coverage analysis.

KIIs highlighted the challenges in data availability. One participant mentioned, “The most reliable approximations of vaccine coverage come from last year’s vaccination data and the door-to-door polio campaign” (K-05). Another added, “Alternative population data is available from OCHA, but it is considered inferior to the more comprehensive and up-to-date polio data” (K-06). This reliance on figures from previous Polio vaccination campaigns is confirmed by our document analysis. In 2021 the SIG vaccinated 134,083 children with Bacillus Calmette–Guérin (BCG). The Polio campaign in the previous year vaccinated a total of 155.378 children under 1. According to third party monitoring, the coverage rate of this polio campaign was 93%. Assuming that the age-distribution of the coverage is equal, this would make the total number of children under 1 in northwest Syria 167.073. Accordingly, the coverage rate for BCG would then be 80.3%. Similar statistics currently being used as coverage data, but these are suboptimal.

Workshop participants echoed these concerns, emphasizing the need for digitalization of medical and vaccination records. A participant remarked, “Paper vaccination cards are often lost, and manual data collection is prone to error. Digital systems are urgently needed” (W-03).

Our analysis indicates that while inclusivity is a stated priority and efforts are made to collect relevant data, the effectiveness of these efforts is limited by significant data availability challenges. Digitalization initiatives are a positive step but require more support and implementation.

### Clear vision with limited participatory strategy development

Field observations showed the SIG’s active involvement in strategic planning, supported by WHO and GAVI. Researchers noted clear mission statements and detailed strategies in the SIG’s multi-year plan, though awareness among partners was limited.

Document analysis confirmed the existence of structured strategic plans but indicated fragmented decision-making processes involving multiple stakeholders, including donors, partners, and the SIG. The SIG was observed to function as a central coordination and mediation platform.

KIIs provided insights into the strategic planning processes, with participants acknowledging sufficient opportunities for input but noting limited participation from partners. One participant stated, “I don’t think the NGOs are participating in finding solutions. Mainly the SIG is doing this. The SIG is doing a good job, so we feel relaxed somehow, so we don’t want to interfere in the system” (K-11). Another added, “It is positive that the implementing partners are only implementing the central plans” (K-06).

Workshop participants supported these findings, expressing trust in the SIG’s strategic planning but also highlighting the lack of engagement from partners in the decision-making process. One participant noted, “The SIG maintains the strategy and the quality of the strategy. In humanitarian crises and the Syrian context, we operate as organizations, but we established a central team” (W-04).

Our analysis suggests that while the SIG has a clear vision and structured strategic plans, the limited participatory strategy development may hinder broader ownership and engagement from all partners.

### Limited transparency

Field observations noted a general perception of the SIG being approachable, but with limited transparency in documentation. Researchers observed that information sharing was mostly internal, with minimal public disclosure.

Document analysis highlighted the lack of an internet presence, financial disclosure, and public availability of strategic plans and annual reports. Information was primarily disseminated through internal reports and meetings, limiting access for external stakeholders.

KIIs revealed a discrepancy between perceived and actual transparency. One participant commented, “A normal Ministry of Health would not separately publish their vaccination results in so much detail” (K-03). Another stated, “Partners funded through the WHO share their financial data with the SIG, but privately funded partners do not” (K-02).

Workshop participants emphasized the need for greater transparency, particularly for stakeholders not directly involved in the SIG’s network. A participant remarked, “It is difficult to obtain information about the topic if one is not part of the network. Only the WHO and the Assistant Coordination Unit (ACU) additionally report on selected aspects of vaccination” (W-01).

Our analysis indicates that while the SIG is considered transparent by partners due to its approachability, the lack of public documentation and financial disclosure limits overall transparency. Enhanced public communication strategies could improve transparency and accountability.

### Accountability and sustainability

Field observations underscored the complex collaboration of stakeholders underpinning vaccine provision, with no single body having legitimate oversight. Filed researchers noted the decentralised structure and reliance on various donors.

Document analysis highlighted the lack of enforcement mechanisms for medical guidelines and protocols. The SIG’s Statement of Principle lacked enforceable standards, leaving de facto power with diverse donors. This patchwork funding approach posed challenges to accountability and sustainability.

KIIs pointed to the absence of a central governance body, with one participant noting, “The donors know that the SIG is not officially on the papers, but they know there is a body called SIG responsible for reaching the target, achieving the indicators, and supervising technically” (K-07). Another participant identified potential risks, stating, “The cut of funds, war, and lack of stability of the security situation. We have the scenario, but we don’t know what will happen” (K-08).

Workshop participants discussed stabilising factors such as the system’s size, decentralized structure, and financial continuity. One participant remarked, “The system grows and becomes a stable system. Everyone is aware of how the system is growing, and this assists the continuity” (W-05).

Our analysis concludes that while there are significant challenges to accountability and sustainability, including fragmented oversight and reliance on diverse donors, stabilizing factors such as decentralization and financial continuity offer some resilience against potential disruptions. Capacity building at district and governorate levels is crucial for ensuring long-term stability and effectiveness.

## Discussion

The primary themes under investigation in this study encompassed the effectiveness and efficiency of the vaccination governance in northwest Syria; its responsiveness, inclusivity, and informed decision-making; its vision and strategy; transparency; and accountability and sustainability.

The management and coordination of vaccination in conflict-affected areas pose significant challenges to effectiveness and efficiency. In regions like northwest Syria, where government control is limited, the discontinuation of routine vaccination services exacerbates these challenges. Comparisons with other conflict-affected areas, such as Myanmar and Somalia, highlight the role of local organizations and international support in filling governance gaps [24, 25]. However, research on vaccination coordination in northwest Syria remains sparse, underscoring the need for a deeper understanding of local structures and operations.

Prior to 2016, the health governance model followed a bottom-up approach, with local entities playing significant roles in vaccination activities. With the establishment of SIG, a hybrid top-down and bottom-up model emerged, shifting the focus to international support and coordination while preserving field connection. This model change reflects the unique challenges of vaccination services in conflict-affected regions and underscores the need for a collaborative approach under the United Nations’ umbrella.

The Syria Immunisation Group (SIG) plays a pivotal role in vaccination governance in northwest Syria, aiming to address these challenges. While SIG has gained internal legitimacy through collaboration with health directorates (HDs) and external legitimacy through collaborating with WHO and UNICEF, concerns regarding accountability and inclusivity persist. The lack of transparency and involvement of partners in strategic planning processes hinder informed decision-making. These finding are in line with a study by Alaref et al. in 2023 which evaluated six governance principles for central quasi-governmental institutions in northwest Syria, including SIG, and found that its legitimacy is fair and requires improvement, scoring 41–60% on a health system governance scale adapted for this paper. Accountability, transparency, effectiveness and efficiency were poor and required significant improvement, scoring 21–40%, while strategic vision was very poor or inactive, scoring 0–20% [26].

Despite having a strategic plan and receiving support from international organisations like the WHO and GAVI, SIG faces contradictions in its effectiveness and efficiency. The transition from emergency task forces to SIG was marked by power dynamics and challenges to local ownership, raising questions about sustainability and integration into national vaccination programs [9]. The potential transition of WHO operations further complicates the future of SIG, posing a key challenge to early recovery in Syria.

These findings raise questions about the future of the SIG body in light of the political and military changes in the region and the constant threat associated with cross-border operations. What would happen if the WHO ceased operations in Gaziantep and moved to Damascus, where a national vaccine program has been in place for decades? In such a scenario, would the SIG continue to carry out its activities in northwest Syria, or would it become a part of the national vaccine program? This is a key challenge for the transition to early recovery in Syria.

## Conclusion

In conclusion, the governance of vaccination in conflict-affected areas of northwest Syria is complex, with multiple stakeholders involved and a lack of a legitimate government to fulfil essential functions. The success of the vaccination program heavily relies on the efforts of the Syria Immunisation Group (SIG), which acts as a trusted mediator between various stakeholders. However, the lack of transparency and accountability hinders the ability to assess the program’s effectiveness and efficiency. This calls for a push towards more localised ownership and transparency, with a hybrid top-down and bottom-up approach that addresses the unique context of conflict settings. Engaging local partners in decision-making and capacity building can improve sustainability and address issues surrounding legitimacy. Moreover, the responsibility to protect public health goes beyond national sovereignty, and the role of international bodies like the WHO becomes crucial in conflict areas. Inaction or delayed action can have catastrophic consequences, as witnessed in Syria with the emergence of diseases like polio and measles. It is essential to implement a structured feedback mechanism and transparent monitoring and evaluation processes to address challenges and foster trust among stakeholders and the community. Ultimately, the findings of this study inform debates around health governance in conflict settings, highlighting the need for more inclusive, transparent, and context-sensitive approaches to ensure the success and sustainability of vaccination programs.

## Electronic supplementary material

Below is the link to the electronic supplementary material.

Supplementary Material 1

## Data Availability

The datasets generated and/or analysed during the current study are not publicly available due to the sensitive nature of the data, but are available from the corresponding author on reasonable request.

## Abbreviations

SIG: Syria Immunisation Group
EWARN: Early Warning and Response Network
EPI: Expanded Programme on Immunization
WHO: World Health Organization
GAVI: Global Alliance for Vaccines and Immunization
OCHA: United Nations Office for the Coordination of Humanitarian Affairs
HDs: Health Directorates
GAVI: Global Alliance for Vaccines and Immunisation
KIIs: Key Informant Interviews
UNICEF: United Nations International Children’s Emergency Fund
