# Supplementary material for: Vaccination governance in protracted conflict settings: the case of northwest Syria
Source: BMC Health Serv Res. 2024 Sep 12;24:1056. doi: 10.1186/s12913-024-11413-1 (PMC11396824; doi:10.1186/s12913-024-11413-1)
Supplement: Supplementary file 1 — Supplementary Material 1 [file 12913_2024_11413_MOESM1_ESM.docx]

**Name:**

**Job title:**

**Tasks:**

**Organisation**

How many centres do you run?

How many donors do you deal with?

How often do you talk to the donors and SIG?

**STRATEGY**

Strategy consists of goals in a few years time, and a clear plan what is needed to get there and who will do what. E.g. number of vaccinations given in one year or new areas covered.

What do you know about the annual plan of the SIG? Have you read it?

What do you think of the 5-year plan? Have you read it?

What strategic planning is done well, and where are the gaps?

What do you think of the following statement: The vaccination leadership has a realistic 5-year plan to guarantee the continuity of the EPI programme, which takes into account worst-case scenarios like the failing of the cross-border resolution.

What do you think of the following statement: The procedures donors require are not always honoured by the SIG, sometimes leading to conflict between implementing partners and the SIG.

**PARTICIPATION**

Participation involves asking the views of all people affected by decisions, and taking them into account. E.g. if working hours are changed, that field staff is consulted, or field staff get to vote about the management.

Does the SIG expect, foster and take into account the input of partners?

What do you think about the extend to which NGOs are making use of the opportunity to give creative input? Do they give valuable input?

What do you think of the degree of input field staff and beneficiaries get?

Do you think any party that should have a say is not getting it?

What do you think of the following statement: The current vaccination-governance is very fragile, because it relies on a small number of irreplaceable key people. Dr. Yasser, perhaps 2-3 of the technical team in SIG. If they leave, the organisation can face chaos and infighting

**TRANSPARENCY**

Transparency means that information from the umbrella organizations, the NGOs and SIG is accessible. Think of finance, procedures, outcomes.

What documents/information does your organization share publicly?

What do you think of the transparency of the SIG?

Is there anything they do not share, and they should share?

Do NGOs share enough information with the SIG? And publicly?

What are reasons not to share certain data?

What are the agreements with the donor about sharing data publicly? For example funding data or outcomes?

To what extent can and do donors influence the degree of transparency of the SIG or NGOs?

What do you think of the following statement: It should be mandatory for all NGOs to publicly publish an annual report regarding their EPI activities, in which they at least share their activities, their funding sources, the money they spent and on what, and the outcomes.

**EFFICIENCY**

Efficiency means the resources are being used optimally.

How well do you think resources are being used in the vaccination strategy?

Do you feel like any resources are being wasted? What are best practices of the use of resources?

What is the degree of corruption you see in the centres?

What is the quality of the field staff in the centres?

What do you think of the amount of paperwork you have to fill out?

REPLY: It would save money if all the proposals and finances were done by the SIG

**INTELLIGENCE**

Intelligence means the degree of information that is available to make well-informed decisions.

What are the greatest challenges regarding the availability of data for key decisions?

What data is being collected systematically?

What is the quality of the data that is available?

**LAW**

Law means the rules and regulations on vaccine quality, accreditation of healthcare providers or standards for facilities.

Are there any laws by local authorities you have to abide by inside of Syria?

And any international laws your organisation needs to abide by?

Which are laws you think should be there but aren’t?

**RESPONSIVENESS**

Responsiveness means the degree to which the vaccination strategy responds to real needs on the grounds.

To what extend does the vaccination strategy cater to real needs on the ground?

Through which tools are vaccine-needs and user satisfaction assessed?

**ETHICS**

Ethics is the consideration of fairness, utility, doing no harm and doing good in the strategy.

Is there an explicit ethics policy?

What are the structures to implement and evaluate such policy?

Are there any issues you’d like to see a more conscious ethical policy on?

**EQUITY**

Equity means that all groups and areas have equal opportunity to access services, even if that means making more effort to reach certain groups or areas.

How well do you think vaccination strategy tries to reach all groups and areas?

Can you mention some best practices?

Is any group or area left behind?

**ACCOUNTABILITY**

Accountibility means that the parties to whom commitments are made (beneficiaries, donors) have the power to make you commit.

Is there any body that the SIG is accountable to?

In case there would be any misconduct in the SIG, which body has the power to approach them about it, and what measures can they take?
